# Adaptation of *Drosophila melanogaster* to Long Photoperiods of High-Latitude Summers Is Facilitated by the *ls-Timeless* Allele

**DOI:** 10.1177/07487304221082448

**Published:** 2022-03-18

**Authors:** Peter Deppisch, Johanna M. Prutscher, Mirko Pegoraro, Eran Tauber, Christian Wegener, Charlotte Helfrich-Förster

**Affiliations:** * Neurobiology and Genetics, Theodor-Boveri Institute, Biocenter, Julius-Maximilians-University of Würzburg, Würzburg, Germany; † Faculty of Science, School of Biological and Environmental Science, Liverpool John Moores University, Liverpool, UK; ‡ Department of Evolutionary and Environmental Biology, Institute of Evolution, University of Haifa, Haifa, Israel

**Keywords:** locomotor activity, *timeless* polymorphism, evening peak, rhythmicity, arrhythmicity

## Abstract

Circadian clocks help animals to be active at the optimal time of the day whereby for most species the daily light-dark cycle is the most important zeitgeber for their circadian clock. In this respect, long arctic summer days are particularly challenging as light is present almost 24 h per day, and continuous light makes the circadian clocks of many animals arrhythmic. This is especially true for the fruit fly, *Drosophila melanogaster*, which possesses a very light-sensitive clock. The blue-light photoreceptor Cryptochrome (CRY) and the clock protein Timeless (TIM) are the light-sensitive components of the circadian clock and are responsible for constant light-induced arrhythmicity even at very low light intensities. Nevertheless, *D. melanogaster* was able to spread from its tropical origin and invade northern latitudes. Here, we tested whether a natural polymorphism at the *timeless* (*tim*) locus, *s-tim* and *ls-tim*, helped adaptation to very long photoperiods. The recently evolved natural allele, *ls-tim*, encodes a longer, less light sensitive form of TIM (L-TIM) in addition to the shorter (S-TIM) form, the only form encoded by the ancient *s-tim* allele. *ls-tim* has evolved in southeastern Italy and slowly spreads to higher latitudes. L-TIM is known to interact less efficiently with CRY as compared with S-TIM. Here, we studied the locomotor activity patterns of ~40 wild *s-tim* and *ls-tim* isofemale lines caught at different latitudes under simulated high-latitude summer light conditions (continuous light or long photoperiods with 20-h daily light). We found that the *ls-tim* lines were significantly more rhythmic under continuous light than the *s-tim* lines. Importantly, the *ls-tim* lines can delay their evening activity under long photoperiods, a behavioral adaptation that appears to be optimal under high-latitude conditions. Our observations suggest that the functional gain associated with *ls-tim* may drive the northern spread of this allele by directional selection.

The ability to anticipate rather than to merely react to daily environmental oscillations strongly contributes to animal fitness (Beaver et al., 2002; Emerson et al., 2008). Organisms achieve this ability thanks to their circadian clocks that allow specific biological activities to occur at precise times of the day (Daan, 2010). The optimal timing of activity largely depends on the latitude at which species live, and this is especially true for ectothermic animals such as insects. In the tropics, it is advantageous to avoid the midday heat and be active during the cooler dawn and dusk or even the night. Yet, at high latitudes, it appears to be better to start activity later during the day and to be active in the afternoon until dusk, when ambient temperatures are higher than in the morning. Indeed, tropical fruit fly species show prominent activity in the morning and evening and keep a siesta in the middle of the day, while high-latitude *Drosophila* species start their main activity later in the day and are most active in the mid to late afternoon until dusk sets in (Menegazzi et al., 2017; Beauchamp et al., 2018). Furthermore, high-latitude species retain some rhythmicity under continuous light, a condition under which tropical flies become arrhythmic (Kauranen et al., 2012; Menegazzi et al., 2017).

The observed differences in the activity pattern of high-latitude flies appear to be caused by specific alterations in the neurochemistry of their clock network (Bahn et al., 2009; Kauranen et al., 2012, 2016; Hermann et al., 2013; Menegazzi et al., 2017; Beauchamp et al., 2018). High-latitude *Drosophila* species belonging to the *virilis-repleta* radiation lack the blue-light sensitive flavoprotein Cryptochrome (CRY) in certain clock neurons that contribute to clock light sensitivity and the neuropeptide Pigment-Dispersing Factor (PDF) in other clock neurons that are responsible for the flies’ morning activity. Furthermore, northern species have more PDF-positive fibers close to the clock neurons that control evening activity. PDF was found to delay the cycling of the evening neurons, leading to a later evening activity under long photoperiods or zeitgeber cycles with long period (Liang et al., 2016, 2017; Menegazzi et al., 2017; Schlichting et al., 2019; Vaze and Helfrich-Förster, 2021).

*D. melanogaster* flies that have invaded northern Europe do not show any differences in the neurochemistry of their clock network in comparison to their tropical siblings. They found other ways to adapt to high latitudes such as polymorphisms in the clock genes *period (per)* and *timeless* (*tim*) (Yang and Edery, 2018; Zonato et al., 2018). For example, natural *per* variations affect the splicing of a short intron in a temperature-depending manner leading to higher *per* mRNA levels and a decrease in the duration of the flies’ siesta at low temperatures (Majercak et al., 1999; Low et al., 2008; Cao and Edery, 2017; Yang and Edery, 2018, 2019). In contrast, variations in *tim* appear to influence mainly the clock’s responses to light. Here, we focus on the latter. A variant of the *timeless* (*tim*) gene affecting the clock’s light sensitivity was discovered many years ago (Rosato et al., 1997). The original *tim* allele encodes a 1398 amino acid long TIM protein (Figure 1a). However, several hundred to thousand years ago, a single guanosine was inserted into the 5ʹ upstream region of *tim.* This resulted in the removal of a stop codon, so that translation could now start from an additional upstream methionine codon leading to the production of a 23 amino acid longer form of TIM (TIM 1421), in addition to the original short form (Rosato et al., 1997; Sandrelli et al., 2007; Tauber et al., 2007; Zonato et al., 2018). The two naturally occurring *tim* alleles are called *s-tim* (encoding only the shorter S-TIM form) and *ls-tim* (encoding both the long L-TIM and short S-TIM form) (Figure 1a).

**Figure 1.**
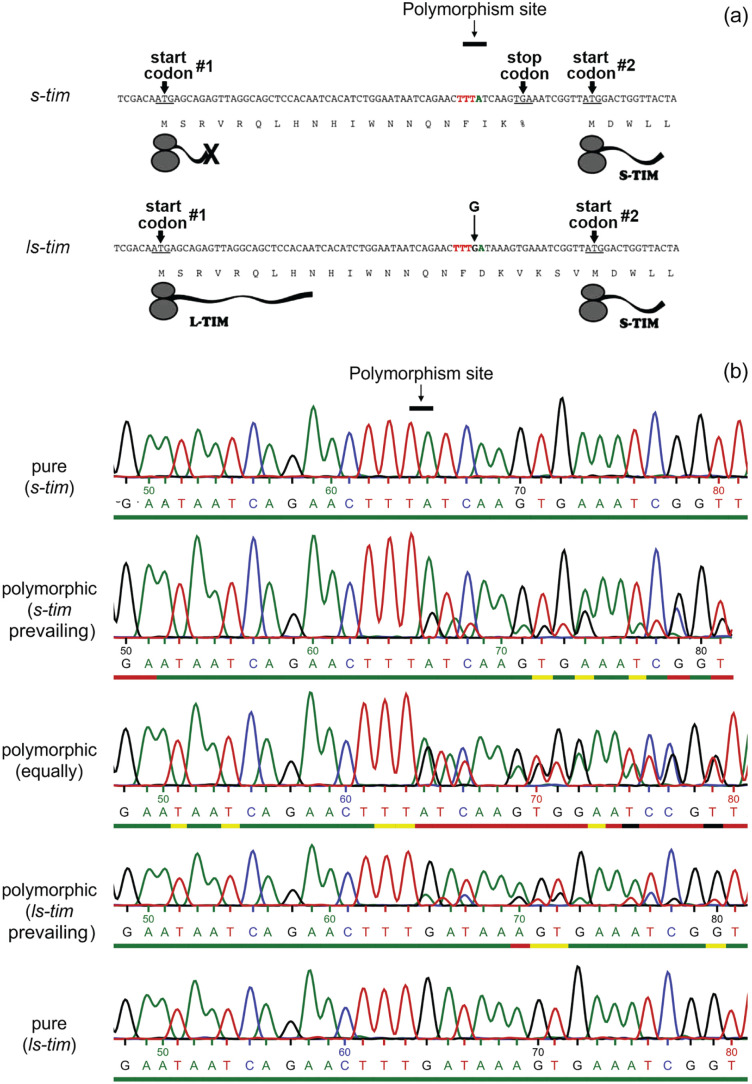
The *tim* polymorphism removes a stop codon and leads to alternative start codons. (a) The N-terminal coding sequences for both *tim* alleles are shown together with their corresponding protein translations. Due to a stop codon (TGA), the original *s-tim* allele generates a 19-residue peptide (starting from the first ATG start codon) and the s-TIM1398 isoform. The G insertion in *ls-tim* after the T triplet (red) leads to a frame shift that removes the stop codon and allows the generation of both the L-TIM1421 and S-TIM1398 isoforms. In addition, there is a C to A transversion 3 base pairs downstream of the G insertion site in the *ls-tim* allele. This single nucleotide polymorphism is linked to the *s-tim/ls-tim* polymorphism (modified after Tauber et al., 2007). (b) Sequenced regions around the *s-tim/ls-tim* polymorphism site are shown. The DNA nucleobases are highlighted in green (adenine), blue (cytosine), black (guanine), and red (thymine). The *s-tim*/*ls-tim* polymorphism sites of different fly strains were amplified with polymerase chain reaction from 10 to 12 pooled individuals and subsequently sequenced. The upper and the lower sequences represent lines with pure prevalence of *s-tim* or *ls-tim* alleles, respectively. The 3 sequence examples in the middle show a shift by 1 nucleobase after the G insertion site, indicating that these strains are polymorphic. The prevalence of one allele in the population was judged visually by the relative height of the adenine and guanine peaks after the G insertion site. If the two peaks were of equal size, we judged the relevant line as “equally mixed”; if one of the two peaks prevailed, we judged the line as *s-tim* or *ls-tim* prevailing, respectively. Also the amplitude at the C/A-polymorphism site, which is linked to either the *s-tim* or *ls-tim* allele, respectively, were considered when categorizing fly strains. The colored bars under the base names describe a quality measure as assessed by the sequencing facility (green: ≥30, yellow: 20-29, red: 10-19, black: 0-9). Mind that the quality decreases because of multiple peaking induced by the polymorphism in mixed lines.

The *ls-tim* isoform may have spread from southeastern Italy (Novoli) to other latitudes due to directed natural selection (Tauber et al., 2007; Zonato et al., 2018). There are 2 putative reasons for directed selection of the *ls-tim* isoform in the north: (1) *ls-tim* flies are less sensitive toward light, due to a weaker CRY-L-TIM interaction and subsequent lower degradation of TIM and CRY by the F-box protein Jetlag (JET) in the proteasome as compared with *s-tim* flies (Sandrelli et al., 2007; Peschel et al., 2009). This may save the *ls-tim* flies from enhanced TIM degradation at northern latitudes with long photoperiods (Sandrelli et al., 2007). (2) *ls-tim* females enter reproductive arrest earlier in response to short days and cold temperatures than *s-tim* females, which clearly favors survival at high latitudes (Tauber et al., 2007; Kyriacou et al., 2008; Pegoraro et al., 2017; Zonato et al., 2017).

Despite the strong evidence for a selective advantage of the *ls-tim* allele at high latitudes, it has so far not been systematically tested whether *ls-tim D. melanogaster* flies can adapt their activity patterns to extremely long photoperiods in a similar way as northern *Drosophila* species with altered clock neurochemistry. This was the aim of the present study. We compared the locomotor activity of 40 *D. melanogaster* lines from Denmark, Britain, Germany, and tropical Africa, 2 lab strains and 7 crosses under different photoperiods (LD 12:12, LD 16:08 and LD 20:04). These lines were either homozygous for *s-tim* or *ls-tim* or they carried a heterogeneous mixture of both alleles (i.e. homozygous *s-tim* and *ls-tim* flies, as well as *ls-tim/s-tim* heterozygotes) (Figure 1b). In addition, we recorded all lines under constant darkness (DD) and constant light (LL) to determine the impact on the *tim* polymorphism on circadian rhythmicity.

## Material and Methods

### Origin, Breeding, and Crossing of the *D. Melanogaster* Lines

All wild-type lines are listed in Table 1. Isofemale lines were established from single fertilized females collected near Market Harbourough/Leicestershire close to Leicester, Britain (52.6° North), a location close to Kopenhagen, Denmark (56.2° North) in autumn 2015, as well as on grape pomace piles at Hubland/Gerbrunn (Hub) and in the orchards of Oellingen (Oel) close to Würzburg (49,5° North) in autumn 2017. Furthermore, we used several African lines. One line stemmed from a population of *s-tim* flies collected in Tanzania (Tan) (3° South) in 2014 (Menegazzi et al., 2017), one line was collected in Zambia (Zam) (15.5° South) and another in Zimbabwe (Zbw) (17.9° South) by John Pool and Bill Ballard, respectively. Four isofemale lines stemming from Accra (Acc) in Ghana (5.5° north) were collected in 2010 and purchased from the National *Drosophila* Species Stock Center (NDSSC) at Cornell. As a comparison, we used 2 lab lines, wild-type Canton-S (*ls-tim)* and wild-type Lindelbach (*s-tim*) (Schlichting et al., 2014) from the Würzburg stock collection. In addition, 3 crossed lines (cross 1, cross 2, and cross 3) were obtained from breeding together a mixture of either pure homozygous *s-tim* or *ls-tim* lines for at least 5 generations. For cross 1, the *s-tim* lines Hub 5, Hub 10, Hub 20, Hub 38, Oel 6 and Oel 9 were bred together. For cross 2, the same was done with the *s-tim* lines Hub 5, Hub 10, Hub 20 and Hub 38, and for cross 3, the *ls-tim* lines Hub 11, Hub 33 and Oel 5 were combined. Furthermore, 2 heterozygous crosses were set up between *s-tim* and *ls-tim* lines from Würzburg and Leicester, respectively, to see whether the F1 offspring shows intermediate phenotypes. The parental lines were selected for the most extreme distance between morning and evening activity peaks under long photoperiods (shortest for *s-tim* lines, longest for *ls-tim* lines). From Würzburg, these were the *s-tim* line Oel 6 and the *ls-tim* line Hub 11 and from Leicester these were the *s-tim* line Lei 38 and the *ls-tim* line Lei 6. We performed 2 crosses per pair that were called cross 4 and 5, and 6 and 7, respectively. In cross 4 and 6, female *s-tim* flies were crossed to male *ls-tim* flies. In cross 5 and 7, female *ls-tim* flies were crossed to male *s-tim* flies (see Table 1).

**Table 1.** *Tim* polymorphism and geographical origin of the *D. melanogaster* lines used in this study (16 German, 13 English, 4 Danish, 7 African, 2 lab lines, and 7 crosses).

Line	*Tim* Polymorphism	Origin
Oel 5	*ls-tim*	Isofemale, Würzburg, 49.6°N/10.0°E
Oel 6	*s-tim*	Isofemale, Würzburg, 49.6°N/10.0°E
Oel 7	polymorphic (*ls-tim* prevailing)	Isofemale, Würzburg, 49.6°N/10.0°E
Oel 9	*s-tim*	Isofemale, Würzburg, 49.6°N/10.0°E
Oel 11	polymorphic (equal mix)	Isofemale, Würzburg, 49.6°N/10.0°E
Hub 5	*s-tim*	Isofemale, Würzburg, 49.8°N/9.9°E
Hub 10	*s-tim*	Isofemale, Würzburg, 49.8°N/9.9°E
Hub 11	*ls-tim*	Isofemale, Würzburg, 49.8°N/9.9°E
Hub 17	polymorphic (equal mix)	Isofemale, Würzburg, 49.8°N/9.9°E
Hub 18	polymorphic (equal mix)	Isofemale, Würzburg, 49.8°N/9.9°E
Hub 20	*s-tim*	Isofemale, Würzburg, 49.8°N/9.9°E
Hub 27	polymorphic (equal mix)	Isofemale, Würzburg, 49.8°N/9.9°E
Hub 28	*s-tim*	Isofemale, Würzburg, 49.8°N/9.9°E
Hub 33	*ls-tim*	Isofemale, Würzburg, 49.8°N/9.9°E
Hub 37	polymorphic (equal mix)	Isofemale, Würzburg, 49.8°N/9.9°E
Hub 38	*s-tim*	Isofemale, Würzburg, 49.8°N/9.9°E
Lei 1	polymorphic (*s-tim* prevailing)	Isofemale, Leicestershire, 52.6°N/1.1°W
Lei 6	*ls-tim*	Isofemale, Leicestershire, 52.6°N/1.1°W
Lei 7	polymorphic (equal mix)	Isofemale, Leicestershire, 52.6°N/1.1°W
Lei 8	*ls-tim*	Isofemale, Leicestershire, 52.6°N/1.1°W
Lei 11	polymorphic (equal mix)	Isofemale, Leicestershire, 52.6°N/1.1°W
Lei 12	polymorphic (*ls-tim* prevailing)	Isofemale, Leicestershire, 52.6°N/1.1°W
Lei 14	*ls-tim*	Isofemale, Leicestershire, 52.6°N/1.1°W
Lei 19	polymorphic (equal mix)	Isofemale, Leicestershire, 52.6°N/1.1°W
Lei 29	*s-tim*	Isofemale, Leicestershire, 52.6°N/1.1°W
Lei 33	polymorphic (*ls-tim* prevailing)	Isofemale, Leicestershire, 52.6°N/1.1°W
Lei 38	*s-tim*	Isofemale, Leicestershire, 52.6°N/1.1°W
Lei 39	polymorphic (s-tim prevailing)	Isofemale, Leicestershire, 52.6°N/1.1°W
Lei 62	*s-tim*	Isofemale, Leicestershire, 52.6°N/1.1°W
Kop 3	*ls-tim*	Isofemale, Kopenhagen, 56.2°N/12.6°E
Kop 10	*ls-tim*	Isofemale, Kopenhagen, 56.2°N/12.6°E
Kop 12	*ls-tim*	Isofemale, Kopenhagen, 56.2°N/12.6°E
Kop 48	*ls-tim*	Isofemale, Kopenhagen, 56.2°N/12.6°E
Tan 1a	*s-tim*	Population,^a^ 3.0°S/37.4°E
Acc 1	*s-tim*	Isofemale, Accra, 5.5°N/0°E
Acc 2	*s-tim*	Isofemale, Accra, 5.5°N/0°E
Acc 3	*s-tim*	Isofemale, Accra, 5.5°N/0°E
Acc 4	*s-tim*	Isofemale, Accra, 5.5°N/0°E
Zam 27	*s-tim*	Isofemale, Zambia, 15.5°S/28.3°E
Zbw 210	*s-tim*	Isofemale, Zimbabwe, 17.9°S/25.8°E
Canton-S	*ls-tim*	Lab stock collection
Lindelbach	*s-tim*	Lab stock collection^b^
Cross 1	*s-tim*	Cross of Hub5, Hub20, Hub38, Oel6, Oel9
Cross 2	*s-tim*	Cross of Hub5, Hub10, Hub20, Hub38
Cross 3	*ls-tim*	Cross of Hub11, Hub33, Oel5
Cross 4	ls/s-tim heterozygotes	F1 of Oel 6 females × Hub 11 males
Cross 5	ls/s-tim heterozygotes	F1 of Hub 11 females × Oel 6 males
Cross 6	ls/s-tim heterozygotes	F1 of Lei 38 females × Lei 6 males
Cross 7	ls/s-tim heterozygotes	F1 of Lei 6 females × Lei 38 males

a. First described in Menegazzi et al. (2017).

b. First described in Schlichting et al. (2014).

*cry*^01^ mutants (*ls-tim)* were used for comparison with the activity patterns of wild-type flies under constant light. All flies were reared and kept on *Drosophila* medium, consisting of 0.8% agar, 2.2% sugar-beet syrup, 8.0% malt extract, 1.8% yeast, 1.0% soy flour, 8.0% corn flour, and 0.3% hydroxybenzoic acid. The flies were maintained under a light-dark (LD) cycle of 12:12 at 18 °C ± 0.2 °C (long-term storage) or 25 °C ± 0.2 °C (short-term breeding before performing the experiments) with a relative humidity (rH) of 60 ± 5%.

### *s-Tim* and *ls-Tim* Genotyping

Before the behavioral experiments were carried out, the flies were examined for their *tim* polymorphism. Genomic DNA was extracted from 10 to 12 flies of each individual line. A 216 bp fragment containing the polymorphism site was amplified using the primers 5ʹ-TACAGATACCGCGCAAATGG-3ʹ and 5ʹ-CAATGCATTCGGGTTGACC-3ʹ in a *Taq*-based polymerase chain reaction (PCR) (JumpStartTM REDTaq, Sigma-Aldrich, Merck, Darmstadt, Germany). The annealing temperature was set to 50 °C and the elongation time was 30 s. The PCR product was purified and sequenced with the Mix2Seq-Kit from Eurofins Genomics (Ebersberg, Germany) using the oligo 5ʹ-CGCAAATGGCTAAGAAGTACC-3ʹ. Sequencing results were analyzed by the purity of the peaks at and downstream the site of the polymorphism (Figure 1b). Fly lines with pure sequencing queues were classified into either *s-tim* or *ls-tim* lines. Lines, in which multiple nucleotide peaks occurred after the polymorphism site (when the sequence was shifted by one base), were categorized as polymorphic. In these lines, the originally collected female must have been either heterozygous for the respective alleles or must have mated with one or several males carrying a different *tim* allele. For these lines, we assessed the prevalence of the different alleles by scoring the height of the nucleotide peaks after the polymorphism site (Figure 1b). Samples, in which the peaks of the 2 nucleotides in question (adenine and guanine) showed a similar height, were judged as equally mixed. Samples, in which the peak of 1 of the 2 nucleotides was higher, were judged as either *s-tim* or *ls-tim* prevailing (Figure 1b). The results of the genotyping are shown in Table 1.

### Locomotor Activity Recording

Fly locomotor activity was monitored using the commercially available *Drosophila* Activity Monitor (DAM)-System of TriKinetics (Waltham, MA, USA) as previously described (Schlichting and Helfrich-Förster, 2015). Male flies, approximately 2 to 6 days old, were transferred under CO_2_-anesthesia into the 5-mm thick tubes containing food (4% sucrose and 2% agar), in which their activity was recorded via detecting the interruptions of an infrared light beam at 1-min intervals. All experiments were conducted in an incubator at a relative humidity of 60%. The incubator was equipped with white neon lamps (TL2004-6; ~50 Hz, 6 W; Pollin Electronic, Pförring, Germany) without additional light filters. All experiments started with several days of entrainment to LD 12:12 cycles, followed by either constant darkness (DD) or constant light (LL), or photoperiods of LD 16:08 and LD 20:04. Light intensity during LD and LL conditions was set to 500 lux.

### Data Analysis

All data were plotted as actograms using ActogramJ (Schmid et al., 2011). Flies that did not survive a recording of 10 days in DD/LL or during the entire LD 16:08 and LD 20:04 photoperiod conditions were excluded from further analysis. For determining the free-running period and power of the rhythm under constant conditions, we used χ^2^-periodogram analysis with a significance level of *p* < 0.05. Flies, in which a rhythm was visible in the actograms for at least 3 days and one or more peak(s) in the periodogram exceeded clearly the significance level, were considered as rhythmic. Rhythm power of these flies was expressed as percentage of the variance. The first 10 days of recording in DD or LL were used for determining period and power of the rhythm. During these 10 days, the periods of the rhythms remained rather stable and complex rhythms (several free-running components) were not observed. Most flies were recorded longer than 10 days under constant conditions, allowing the judgment of period changes and the occurrence of complex rhythmicity. In cases where we could see such rhythm characteristics in the actograms, we additionally performed periodogram analysis for the entire recording period.

From the average rhythm period and power of each fly line, we calculated the average period and power for all lines stemming from one region (tropical Africa, Germany, England, Denmark), as well as the average period and power for all fly lines showing a particular *tim* polymorphism (*s-tim* including the *s-tim* prevailing lines, *ls-tim* including the *ls-tim* prevailing lines, equal mix of *s-tim* and *ls-tim*). Similarly, we determined and compared the percentages of rhythmic flies for lines stemming from different regions and with different *tim* polymorphisms.

**Figure 2.**
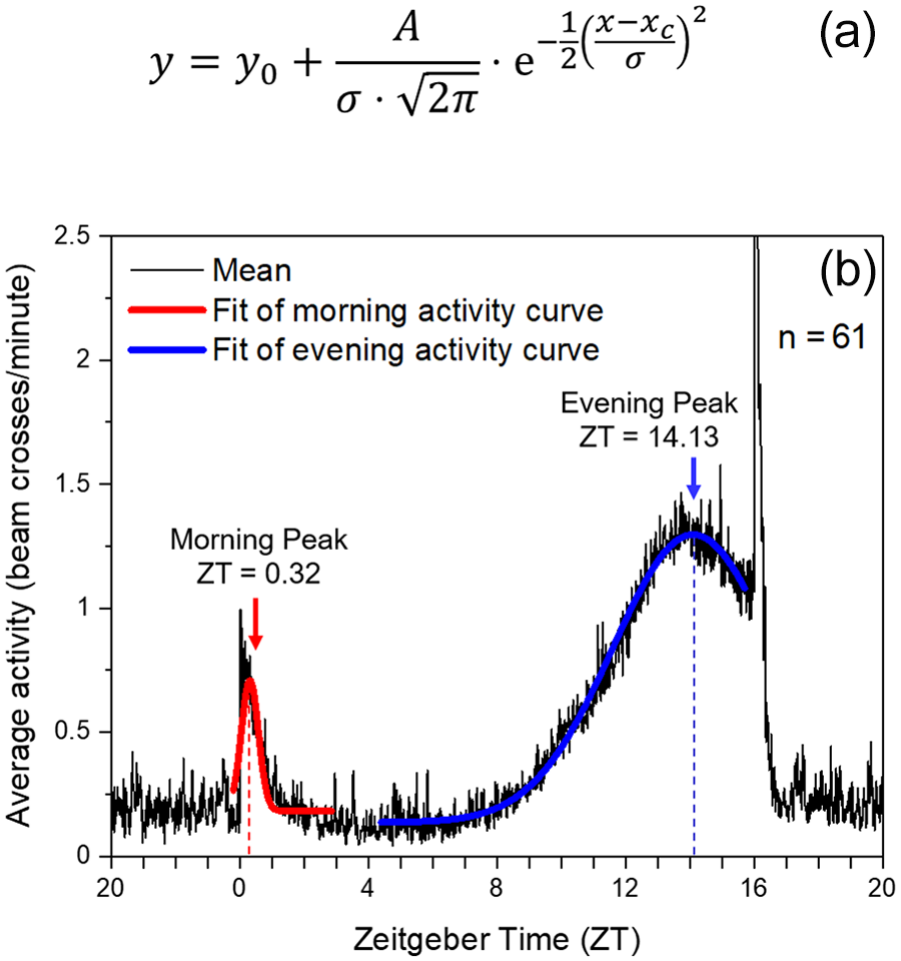
Morning (M) and evening (E) peak analysis under LD 16:08. The average activity profile of each fly line was fitted with Origin’s Quick Fit Gauss tool that applied the function shown in A to selected areas of the activity profile shown in B (area used for calculation of the M peak shown in red and that for the E peak in blue). Abbreviations: *y* = activity (y-axis variable); *y_0_* = offset; *A* = amplitude; σ = standard deviation; *x* = zeitgeber time (ZT; x-axis variable); *x_c_* = peak position (peak ZT).

Under LD 12:12, LD 16:08 and LD 20:04, we calculated average activity profiles for each isofemale line using the last 5 days of each light condition as described previously (Schlichting and Helfrich-Förster, 2015). The maxima of morning and evening activity were calculated using the graphical average activity profiles for each fly or for each fly line. Morning and evening activity maxima of each single fly was determined as described in Schlichting and Helfrich-Förster (2015). For the fly lines, a Gaussian function (Quick Fit Gauss tool) implemented in OriginPro (OriginLab Northampton, MA, USA) was used to fit the curves and determine the relevant morning and evening activity peaks (Figure 2). In some cases, particularly under LD 16:08, the activity was smoothed prior to fitting (using a moving average 11 filter). This helped to distinguish the evening peak from the closely located lights-off startle response. From the determined times of M and E peaks, the distance between the two values (Δψ_M, E_) was calculated and given in hours for each line.

From the average activity profile of each fly line, we calculated average activity profiles for all lines from one region (tropical Africa, Germany, England, Denmark) as well as average activity profiles for all lines showing a certain *tim* polymorphism (according to the definition above).

### Statistical Analysis

The Kolmogorov-Smirnov test was used to test for normal distribution of all values. Subsequently, 1- or 2-way analyses of variance (ANOVAs) were performed to determine whether periods in DD and LL depended significantly on the *tim* polymorphism and/or on the geographical origin of the lines. Similarly, we determined whether Δψ_M, E_ depended on *tim* polymorphism and geographical origin of the lines. Afterward, we performed a Bonferroni post hoc test to reveal significant differences between individual fly lines. When the data were not normally distributed, we used a Kruskal-Wallis test. To test for a correlation between *tim* polymorphism and geographical location, as well as between *tim* polymorphism and circadian rhythmicity under DD and LL conditions, data were arranged in contingency tables for χ^2^ analyses (Zar, 1984).

## Results

### Geographical Distribution of *s-Tim* and *ls-Tim* Flies

**Figure 3.**
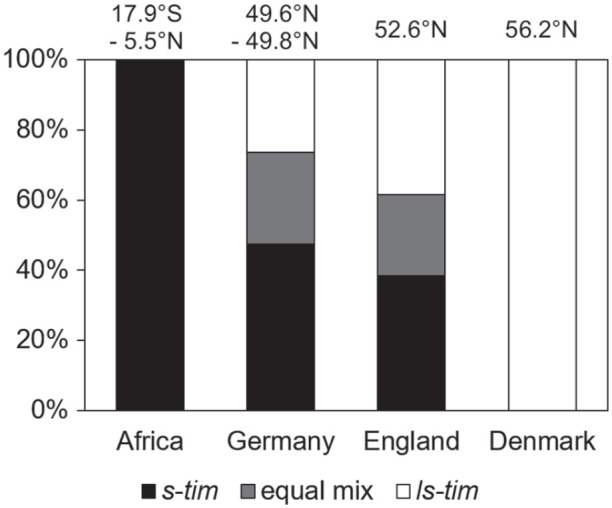
Distribution of *ls-tim* and *s-tim* flies in *D. melanogaster* lines from tropical Africa, Germany, England, and Denmark. The distribution was significantly different at the 4 locations (χ^2^ = 17.81; *p* = 0.007; *df* = 6; *n* = 40).

Of the 40 wild *D. melanogaster* lines, 19 were completely or primarily *s-tim*, 8 carried an equal mix of *s-tim* and *ls-tim* alleles, and 13 were completely or primarily *ls-tim*. Interestingly, the distribution of the *s-* and *ls-tim* lines was significantly different at the different latitudes (Figure 3). As expected, all African lines were *s-tim*. The German and English lines contained a mixture of *s-tim* and *ls-tim* flies with a tendency for more *ls-tim* toward the north, and all Danish lines were *ls-tim*.

### Rhythmic Behavior Under Constant Darkness (DD)

**Figure 4.**
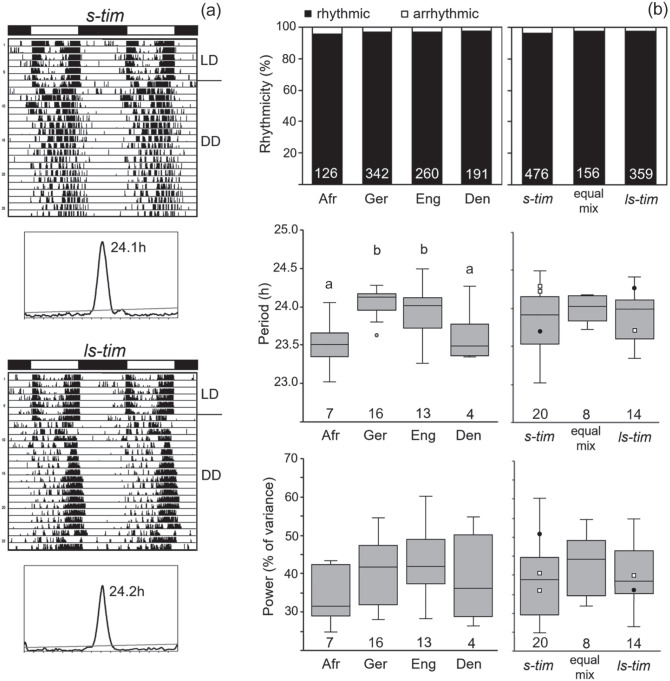
Rhythm parameters of the different wild lines under constant darkness (DD). (a) Typical actograms and periodograms under DD of 2 flies with different *tim* polymorphism that were recorded under light-dark cycles (LD 12:12) and constant darkness (DD). The 2 flies stem from lines collected in Würzburg (Germany) that are *s-tim* and *ls-tim*, respectively. Under LD, the flies show bimodal activity patterns with morning and evening activity, separated by a siesta. Under DD, they free-run with a period close to 24 h. (b) Rhythmicity, circadian period, and power of the different lines from the different geographical locations (left) and with different *tim* genotype (right). Neither location (Den: Denmark, Eng: England, Ger: Germany, Afr: Africa) nor *tim* genotype affected the percentage of rhythmic flies (location: χ^2^ = 0.80; *p* = 0.849; *df* = 3; *n* = 919; *tim* genotype: χ^2^ = 1.706; *p* = 0.426; *df* = 2; *n* = 991). The number of tested flies is shown in white letters at the bottom of the columns. Period and power of the free-running rhythms are indicated as box plots including outliers for the different lines. Filled black points indicate power and period of the lab strains Lindelbach (*s-tim*) and Canton-S (*ls-tim*), respectively. Small open squares depict period and power of the crosses 1 to 3 (see Table 1). The periods were significantly different between the locations as indicated by different letters, ANOVA: *F*_(3,36)_ = 6.824, *p* < 0.001; Post hoc Bonferroni test: Ger × Afr *p* < 0.01, Eng × Afr: *p* < 0.05. In contrast, the periods did not differ significantly between the timeless polymorphisms, ANOVA: *F*_(2,39)_ = 0.413, *p* = 0.498. The number of tested lines is shown at the bottom of the box plots. The number of tested flies per line is given in Supplementary Table S1.

Overall, 962 flies of the different wild lines plus 72 lab flies (Canton-S and Lindelbach) were recorded under LD 12:12 and DD conditions (Suppl. Table S1). Under LD 12:12, all flies exhibited bimodal activity patterns with morning (M) and evening (E) activity, which were separated by a siesta. Figure 4a shows examples of 2 flies from Germany carrying s-*tim*-and ls-tim, respectively. Under DD conditions, the vast majority of the flies was rhythmic and free-ran with a period close to 24 hours (Suppl. Table S1; Figure 4). Neither the *tim* polymorphism nor the geographical location influenced the percentage of rhythmic flies (Figure 4b). Free-running periods were quite variable in the different lines and clearly depended on the geographical location (Denmark, England, Germany, and tropical Africa) (Figure 4b). Nevertheless, periods were independent of the *tim* polymorphism (Figure 4b). The periods of the 2 lab lines (black points in the box plots of Figure 4b) and of crosses 1 to 3 (open squares in the box plots of Figure 4b) with different *tim* polymorphism nicely illustrate this. While the *s-tim* Lindelbach line possessed a short period of 23.7 h, the *ls-tim* Canton-S line had a long period of 24.4 h. In contrast, the *s-tim* crosses 1 and 2 had long periods of 24.2 and 24.3 h, respectively, while the *ls-tim* cross free-ran with a short period of 23.7 h (Figure 4b and Suppl. Table S1). Rhythm power was also quite variable, but neither dependent on location nor on *tim* polymorphism (Figure 4b).

### Rhythmic Behavior Under Constant Light (LL)

**Figure 5.**
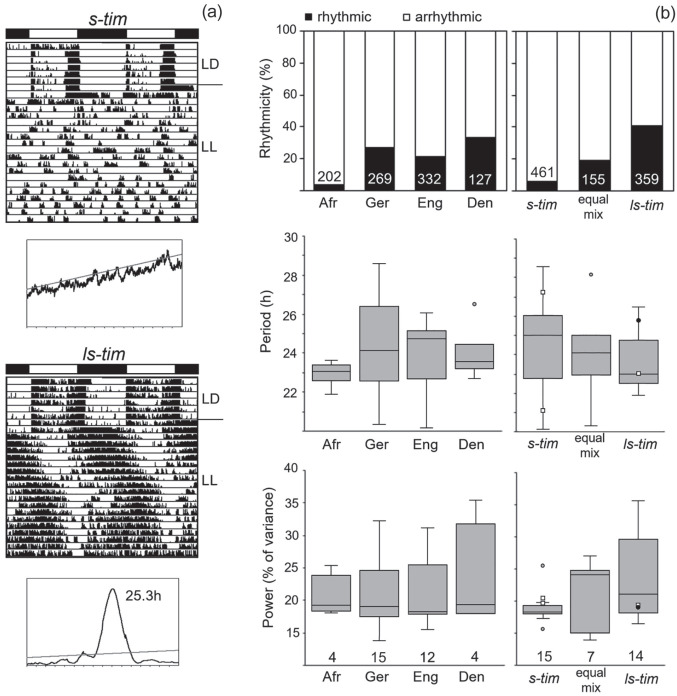
Rhythm parameters of the different wild lines under constant light (LL). (a) Typical actograms and periodograms under LL of 2 flies with different *tim* polymorphism that were recorded under light-dark cycles (LD 12:12) and constant light (LL). The 2 flies stem from lines collected in Würzburg (Germany) that are *s-tim* and *ls-tim*, respectively (same arrangement as in Figure 4a). Under LL, the *s-tim* fly was completely arrhythmic, while the *ls-tim* fly still showed significant rhythms. (b) Rhythmicity, period, and power of the different lines in dependence of their geographical location (left) and their *tim* polymorphism (right). Both location (Den: Denmark, Eng: England, Ger: Germany, Afr: Africa) and *tim* polymorphism affected the percentage of rhythmic flies (location: χ^2^ = 55.18; *p* < 0.001; *df* = 3; *n* = 975; *tim* polymorphism: χ^2^ = 146.13; *p* < 0.001; *df* = 2; *n* = 990). The number of tested flies is shown in white letters at the bottom of the columns. Periods and powers of the free-running rhythms are depicted as box plots for the different lines (labeling as in Figure 4). Neither period nor power were significantly dependent on *tim* polymorphism, ANOVA; *F*_(2,33)_ = 1.006; *p* = 0.377, nor on location, ANOVA; *F*_(2,31)_ = 0.553; *p* = 0.65. The number of tested lines with rhythmic flies is shown on the bottom. The number of rhythmic flies per line is given in Supplementary Table S2.

Overall, 975 flies of the different wild lines plus 45 lab flies (Canton-S and Lindelbach) and 32 *cry*^01^ mutants were recorded under LD 12:12 and LL conditions (Suppl. Table S2). Again, all flies exhibited bimodal activity patterns under LD 12:12. When released into LL (500 lux), most flies became arrhythmic (Figure 5). Nevertheless, the visual inspection of the actograms revealed differences between the *s-tim* and *ls-tim* flies (shown for individual flies from Germany in Figure 5). While virtually all *s-tim* flies became arrhythmic, a significant higher proportion of completely or predominantly *ls-tim* and *s-tim/ls-tim* equally mixed flies remained rhythmic, at least for several days after the transfer to LL. This was also demonstrated in the 2 lab strains. While all *s-tim* Lindelbach flies became immediately arrhythmic after transfer to LL, 53.3% of *ls-tim* Canton-S flies retained rhythmicity for at least 3 days in LL (Suppl. Table S2). Of the *s-tim* flies from crosses 1 and 2, only 6.3% retained some rhythmicity under LL, while in the *ls-tim* flies of cross 3, rhythmicity was detected in 76.9% (Suppl. Table S2). None of the wild *s-tim* lines showed a percentage of rhythmic flies above 15%, but there were several *ls-tim* lines and lines carrying an equal mix of *ls-tim* and *s-tim*, in which more than 50% of the flies showed rhythmicity (e.g., Oel 5, Oel 7, Hub 27, Lei 14, Kop 3; Suppl. Table S2).

**Figure 6.**
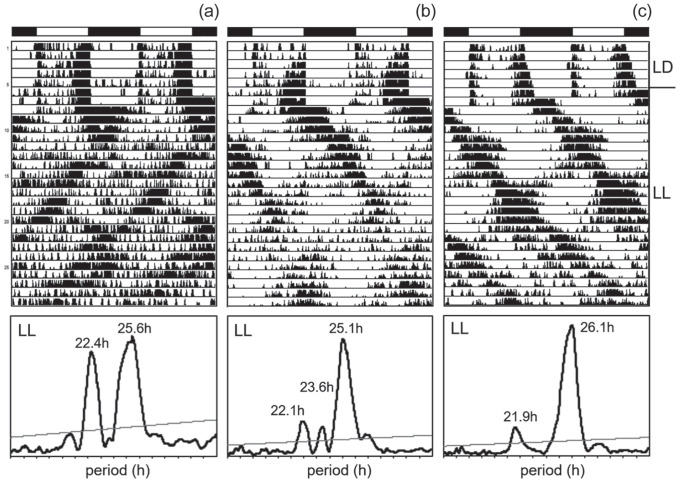
Locomotor activity of *ls-tim* flies in LL. The wild fly in (a) is derived from Würzburg and the one in (b) from Leicester. The fly in (c) is a *cry*^01^ mutant recorded in parallel. Here, periodogram analysis was performed for the entire 22 days in LL and revealed several free-running components that are also visible in the actograms. Abbreviation: LL = constant light.

Nevertheless, not every *ls-tim* line showed this high rhythmicity. For example, of the 4 Danish lines, only 1 line was highly rhythmic, while the others had a rather low percentage of rhythmicity. In all the rhythmic flies, the period of the free-running rhythm was more variable, and the rhythm power was lower as compared with DD conditions (Suppl. Table S2, Figure 5b). As evident from the actogram of the *ls-tim* fly shown in Figure 5a, period often changed during the recording time, and in many cases several similarly free-running components could be detected in the acto- and periodograms of the longer recordings (Figure 6a and 6b). This situation strongly resembled the complex activity pattern of *cryptochrome* (*cry*^b^ or *cry*^01^) mutants under LL (Yoshii et al., 2004; Rieger et al., 2006; Dolezelova et al., 2007), which were mostly composed of 2 free-running components as shown here for 1 *cry*^01^ mutant fly (Figure 6c).

### Activity Patterns Under LD 12:12

**Figure 7.**
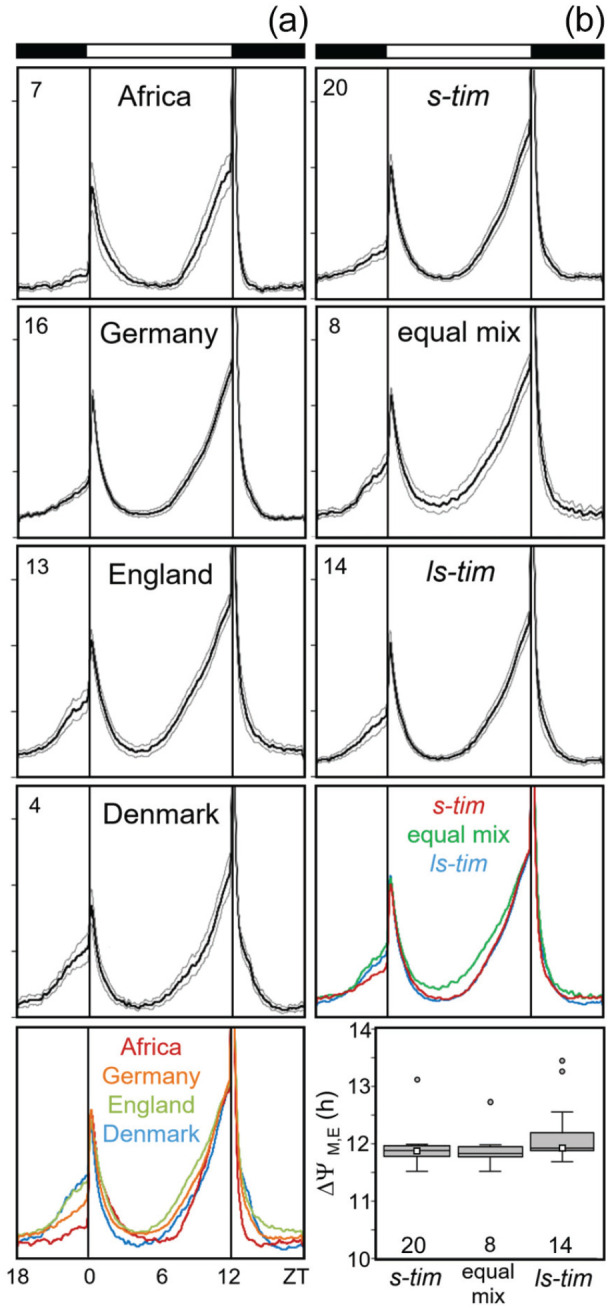
Average daily activity profiles of all lines under LD 12:12. Activity profiles are separately shown for different locations (a) and *tim* alleles (b). Means are indicated by thick lines with the standard errors of the mean as thin lines above and below, respectively. Number of lines is shown in the left top of each diagram. The mean profiles of A and B are compared as colored curves on the bottom, respectively (normalized to the evening maximum). Although the activity profiles of the lines are slightly different between flies of the 4 locations (bottom left), they are rather similar between the flies carrying different *tim* alleles (right). In addition, the timing of M and E peaks did not depend on the *tim* polymorphism: the phase difference between M and E peaks (Δψ_M, E_) was similar in flies with different *tim* polymorphism (bottom right). The number of tested flies per line is given in Supplementary Table S3.

As already mentioned, all flies exhibited bimodal activity patterns with M and E activity bouts around lights-on and -off. The analysis and comparison of average activity profiles revealed that the timing of M and E peaks was similar among the different wild lines, although some differences in the overall activity pattern occurred between the lines from the different locations (Figure 7), which appeared to be independent of the *tim* polymorphism. No significant differences in the timing of M and E activity were observed between *s-tim and ls-tim* flies (Kruskal-Wallis test followed by a post hoc Bonferroni test: *p* = 0.287; Figure 7b, bottom). The same was true for the 2 lab lines and the flies from crosses 1 and 2. The calculated values of their Δψ_M, E_ were very similar and not distinguishable from each other in the box plots (Figure 7b).

### Activity Patterns Under LD With Long Day Lengths (Photoperiods)

**Figure 8.**
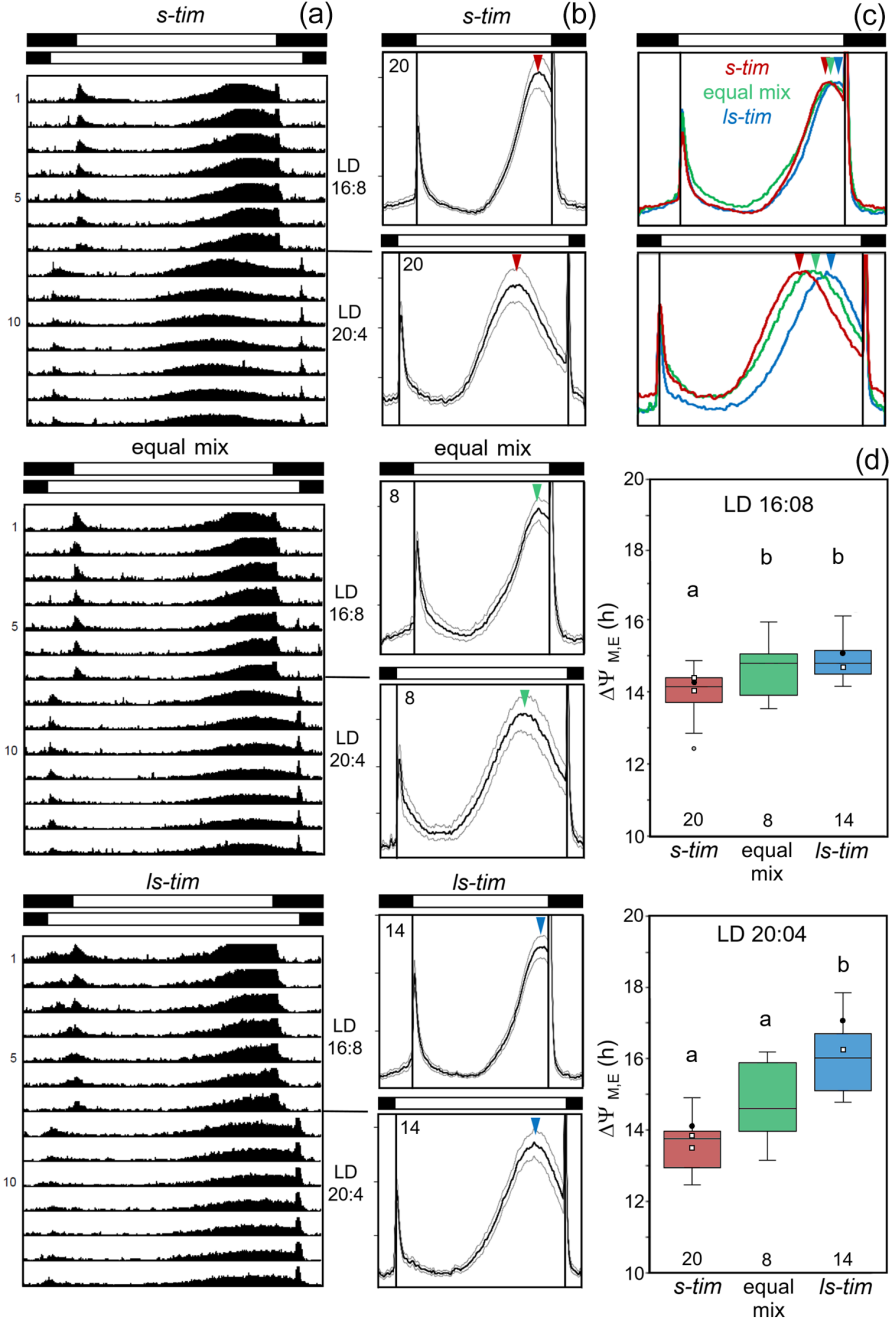
Actograms, activity profiles, and phase distance between morning and evening maxima in flies with different *tim* alleles under long photoperiods. (a) Average actograms for 3 German lines with different *tim* alleles, under LD 16:08 and LD 20:04 (each actogram shows the average activity of 60 flies). (b) Average activity profiles for all lines with *s-tim, ls-tim*, or an equal mix of *s-* and *ls*-*tim* under LD 16:08 and LD 20:04. Numbers in the left upper corners indicate the number of investigated lines and colored arrowheads point to the maxima of evening activity. (c) Comparison of the activity profiles of *s-tim, ls-tim* or mixed *s-* and *ls*-*tim* under LD 16:08 and LD 20:04. Curves are normalized to the height of the evening maxima. (d) Boxplots indicating the phase differences between morning and evening peaks (Δψ_M, E_) under LD 16:08 and LD 20:04. Filled black points indicate Δψ_M, E_ of the lab strains Lindelbach (*s-tim*) and Canton-S (*ls-tim*) and small open squares those of the crosses 1 to 3 (see also Suppl. Table S3). Significant differences are indicated by different letters. The number of tested flies per line is given in Supplementary Table S3. Abbreviation: LD = light-dark.

Overall, 2450 flies of the different wild lines plus 119 lab flies (Canton-S and Lindelbach) were monitored under long photoperiods (LD 16:08, LD 20:04) (Suppl. Table S3). The flies were first recorded for few days under LD 12:12 and then the photoperiod was extended to 16 h. After 7 days, the photoperiod was further extended to 20 h and the flies were recorded for another 7 days. As can be seen in the average actograms of 3 Würzburg lines with different *tim* alleles (Figure 8a), the timing of evening activity depended on the *tim* polymorphism, ANOVA for LD 16:08: *F*_(2,38)_ = 8.381; *p* < 0.001; ANOVA for LD 20:04: *F*_(2,38)_ = 31.249; *p* < 0.001. *ls-tim* flies had a significant later phase of evening activity than *s-tim* flies. This was already significant under 16:08 photoperiods, where post hoc Bonferroni test revealed a significant difference between *s-tim* and *ls-tim* populations (*p* < 0.01). However, the largest difference emerged under extremely long photoperiods (LD 20:04). Evening activity of *ls-tim* flies almost tracked lights-off, while that of *s-tim* flies did not, but occurred at mid-afternoon under LD 20:04 (Figure 8b and 8c). Here, post hoc Bonferroni test revealed a highly significant difference between *s-tim* and *ls-tim* populations (*p* < 0.001). Since morning activity remained around lights-on for all lines under both photoperiods, the phase difference Δψ_M, E_ between morning and evening activity was significantly larger in *ls-tim* flies than in *s-tim* flies, while flies carrying a mixture of both alleles were in an intermediate range (Figure 8d). Flies from the 2 lab lines and from crosses 1 to 3 behaved as the other *s-tim* and *ls-tim* lines: *s-tim* flies showed a small and *ls-tim* flies a large Δψ_M, E_ (Figure 8d, Suppl. Table S3).

### Correlation Analysis and Crosses Between *s-Tim* and *ls-Tim* Lines

**Figure 9.**
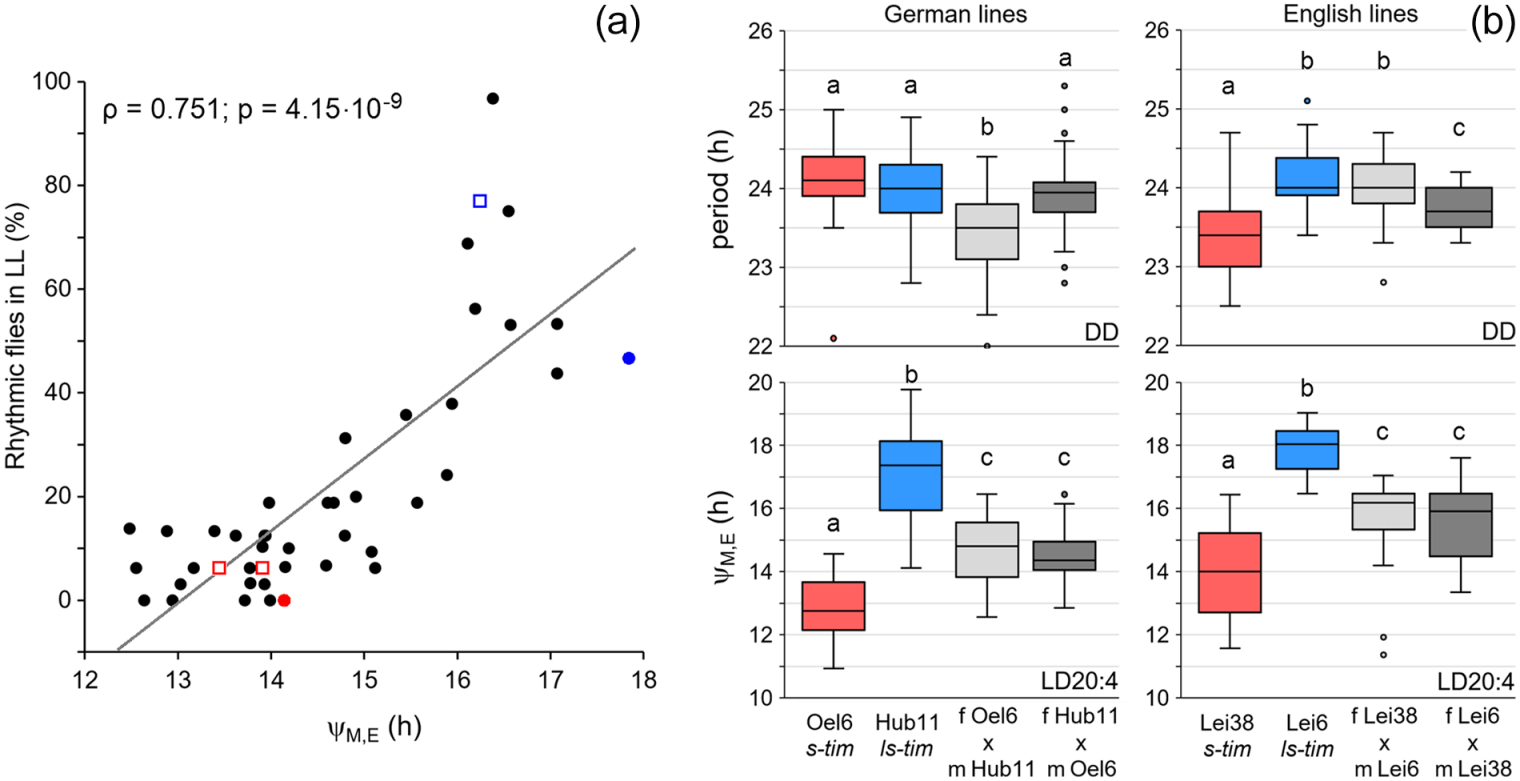
Correlation analysis and results from crosses between *s-tim* and *ls-tim* lines. (a). Correlation analysis between rhythmicity under constant light (LL) and phase distance between morning and evening peaks (Δψ_M, E_). A strong correlation was detected (ρ: Spearman’s correlation coefficient). Red and blue points mark the values for the lab lines Lindelbach and Canton-S, respectively, while red and blue open squares mark the values of crosses 1 and 3 (*s-tim* lines in red and the *ls-tim* line in blue). (b) Free-running periods under constant darkness (DD) and Δψ_M, E_ under long photoperiods (LD 20:4) of about 30 males, each, stemming from selected wild German and English *s-tim* and *ls-tim* lines and their male F1 offspring. The box plots from the male parental generation are colored (red: *s-tim*, blue: *ls-tim*) while the box plots from the male F1 generation are shown in gray (light gray: female *s-tim* crossed to male *ls-tim*; dark gray: female *ls-tim* crossed to male *s-tim*). Significant differences are indicated by different letters above the box plots (ANOVA followed by Bonferroni post hoc tests: *p* < 0.01 for period; *p* < 0.001 for Δψ_M, E_).

Since rhythmicity under LL conditions and a large phase distance between morning and evening activity appeared to be typical for *ls-tim* lines, we tested whether these are correlated. We found a strong and significant positive correlation between the percentage of rhythmic flies and Δψ_M, E_, suggesting that both phenotypes are linked (Figure 9a).

To test further whether the *tim* polymorphism is a likely cause for the delayed evening activity, we performed crosses between selected *s-tim* and *ls-tim* lines, which showed large differences in the distances between morning and evening activity under long photoperiods (LD 20:4) and tested the F1 generation. We chose 2 lines from Germany (Oel 6 and Hub 11) with similar free-running periods and 2 lines from England (Lei 38 and Lei 6) with significantly different free-running periods under DD conditions (Figure 9b, Suppl. Table S1). While the free-running periods of the F1 males were not always in-between the free-running periods of the male parents, the distances between morning and evening activity of the F1 males were intermediate to those of the parental generation (Figure 9b). Furthermore, the F1 free-running periods depended on the direction of the cross (Figure 9b), while the phase distances between morning and evening activity did not (Figure 9b). This speaks for a X-chromosomal inheritance of the free-running period and an autosomal inheritance of the distance between morning and evening activity, which fits well with the location of the *per* gene on the X-chromosome and the *tim* gene on the second chromosome.

Altogether, the results from the crossing experiments underline our conclusion that the *tim* polymorphism affects the phase of evening activity under long photoperiods but not the free-running period under DD.

## Discussion

### Evidence for Directed Selection of the *ls-Tim* Allele

Previous work indicates that the *ls-tim* variant of *timeless* originated in southeastern Italy and then spread, and probably is still spreading, by directional selection (Tauber et al., 2007; Zonato et al., 2018). While the original study of Tauber et al. (2007) estimated the age of *ls-tim* to several thousand years, Zonato et al. (2018), using linkage disequilibrium and coalescent-based approaches, found that it is most probably only several hundred years old (300 to max 3000). This relatively young age may explain why *ls-tim* has not yet gone to fixation.

Compared with *s-tim* lines, flies carrying the new *ls-tim* allele do not show any differences in their period and rhythm power under constant darkness, nor do they have impairments in temperature compensation (Tauber et al., 2007). Thus, their circadian clock appears to work normally, a finding that we confirm with our study. Similarly, we found no influence of the *tim* polymorphism on the activity pattern and timing of morning and evening activity peaks under LD 12:12 conditions typical of tropic regions. The only significant differences between *s-tim* and *ls-tim* flies occur under long photoperiods and constant light conditions typical for high latitudes. Again, this is consistent with previous findings showing photoperiod-related phenotypes of *ls-tim* flies, which are adaptive in seasonal environments (Tauber et al., 2007; Sandrelli et al., 2007). The *ls-tim* allele attenuates the photosensitivity of the circadian clock by causing decreased dimerization of the L-TIM protein isoform to the circadian photoreceptor CRY (Sandrelli et al., 2007). This reduced interaction results in a more stable TIM product that is degraded more slowly by light. Therefore, long photoperiods and constant light are sensed less effectively by the circadian timing system, resulting in an earlier onset of reproductive dormancy in autumn. This is an adaptive advantage in the north, where winter onset occurs early. Thus, it is very likely that the earlier onset of reproductive dormancy serves as a driving force for the natural selection of the *ls-tim* allele. Very recently, an additional positive selection drive for *ls-tim* was proposed by Lamaze et al. (2021), who found that *ls-tim* flies were able to synchronize their activity rhythms to temperature cycles in constant light and simulated natural-like long summer days of Finland, while *s-tim* flies were unable to do so under such conditions. In the present study, we show that the *ls-tim* allele facilitates the adaptation of flies to constant light and long photoperiods even under constant temperature.

### Rhythmicity of *ls-Tim* Flies Under Continuous Light (LL)

So far, no direct effects of the *ls-tim* polymorphism alone on rhythmicity under LL conditions have been reported. The *ls-tim* polymorphism only contributed to higher LL rhythmicity in combination with other genetic predispositions (Peschel et al., 2006; Sandrelli et al., 2007; de Azevedo et al., 2020). Here, we found that a significant higher proportion of *ls-tim* and *s-tim/ls-tim* mixed genotypes remained rhythmic under LL as compared with *s-tim* flies, that virtually all became arrhythmic. In several *ls-tim* lines, more than 50% of the flies showed significant rhythms in LL. Again, this is consistent with the above-mentioned attenuated photosensitivity of the circadian clock caused by the decreased dimerization of L-TIM with CRY. While in *s-tim* wild-type flies TIM is permanently degraded after light-induced interaction with CRY eventually leading to the arrest of the clock (Stanewsky et al., 1998; Ceriani et al., 1999; Emery et al., 2000; Rosato et al., 2001; Busza et al., 2004), this does not happen in flies lacking CRY and is expected to be diminished in *ls-tim* flies with decreased CRY-TIM dimerization. Consequently, *ls-tim* flies should retain some rhythmicity under LL as do *cry* mutants, and this was exactly what we observed. As found in *cry* mutants (Yoshii et al., 2004; Rieger et al., 2006; Dolezelova et al., 2007), the LL rhythms of *ls-tim* flies were often composed of 2 main free-running components. This behavior can be explained by the 2-oscillator model originally proposed by Pittendrigh and Daan (1976) predicting that light from the eyes speeds up morning and slows down evening oscillators, which is important for seasonal adaptation (reviewed in Helfrich-Förster, 2009; Yoshii et al., 2012).

Nevertheless, not all *ls-tim* lines showed a clear rhythmicity under LL, indicating that other factors besides TIM contribute to light sensitivity and affect LL rhythmicity. The F-box protein JET is a likely candidate (Koh et al., 2006; Peschel et al., 2006, 2009). JET associates with TIM and CRY in a light-dependent fashion and promotes the ubiquitination and degradation of both proteins in the proteasome, while the affinity of TIM for CRY and JET determines the sequential order of their degradation (Peschel et al., 2009). In *ls-tim* flies, the affinity between TIM and CRY is reduced, leading to a preferential light-dependent association of JET with CRY and a following CRY degradation, while TIM remains more stable. This may then support LL rhythmicity of *ls-tim* flies. However, Peschel et al. (2006) demonstrated that LL-rhythmicity is only increased when mutant alleles of *jet*—*jet*^c^ or *jet*^r^—are combined with *ls-tim*. These variants have so far only been identified in lab but not in any worldwide collected wild fly lines (Peschel et al., 2006). Nevertheless, we cannot rule out the possibility that other changes have occurred in the *jet* gene of certain wild lines, which might affect the JET protein and ultimately the degradation of TIM and LL rhythmicity.

Other factors affecting the LL rhythmicity of flies in combination with *ls-tim* are genes coding for the light-input factor “Quasimodo” and the Na^+^, K^+^, Cl^-^ cotransporter NKCC (Chen et al., 2011; Buhl et al., 2016) as well as for enzymes involved in glutamate metabolism or metabotropic glutamate receptors (de Azevedo et al., 2020). Mutations in all these genes led to high rhythmicity under LL when the flies were additionally homo- or heterozygous for *ls-tim.*

Most interestingly, the genes coding for JET and proteins involved in glutamate signaling are located together with the *tim* gene on chromosome 2, on which Adewoye et al. (2017) have identified a major Quantitative Trait Locus (QTL) associated with variation in circadian photosensitivity. This opens the possibility that polymorphisms in these genes and other loci in this genomic region may contribute to circadian photosensitivity.

### Late Evening Activity of *ls-Tim* Flies Under Long Photoperiods (LD 16:08 and LD 20:04)

The ability to delay the evening activity under long photoperiods is thought to be adaptive at high latitudes, because at this time of the day, temperatures are warmer than in the morning. Many high-latitude species show a broad activity bout during the afternoon and evening that lasts until dusk even under very long photoperiods (Menegazzi et al., 2017; Beauchamp et al., 2018). The main factors that delay the maximum of evening activity are light through the compound eyes, increased neuropeptide signaling of Pigment-Dispersing Factor (PDF), and reduced levels of CRY. The *Drosophila* high-latitude species studied lack CRY in certain clock neurons and have a high number of PDF-positive neurites close to the clock neurons that control evening activity (Hermann et al., 2013; Menegazzi et al., 2017; Beauchamp et al., 2018). Also in *D. melanogaster*, PDF delays evening activity, while CRY rather advances it, and both do so by acting on or in the evening neurons, respectively (Liang et al., 2016, 2017; Menegazzi et al., 2017; Kistenpfennig et al., 2018; Schlichting et al., 2019; Vaze and Helfrich-Förster, 2021). Consequently, the general loss of CRY (or the loss of CRY in the evening neurons alone) leads to a late maximum of evening activity under long photoperiods (Rieger et al., 2003; Kistenpfennig et al., 2018). This is exactly what we found here in *ls-tim* flies. As mentioned above, the affinity between TIM and CRY is reduced in *ls-tim* flies, which leads to a preferential light-dependent association of JET with CRY followed by CRY degradation (Peschel et al., 2009). Thus, *ls-tim* flies are expected to have lower CRY levels than *s-tim* flies, which can explain why they have a later evening activity than *s-tim* flies, especially under long photoperiods when light is present for a long time.

Altogether, our results strengthen the hypothesis that the *ls-tim* allele is associated with selective advantages at high latitudes allowing range expansion to the North.

## Supplemental Material

sj-docx-1-jbr-10.1177_07487304221082448 – Supplemental material for Adaptation of *Drosophila melanogaster* to Long Photoperiods of High-Latitude Summers Is Facilitated by the *ls-Timeless* Allele Click here for additional data file. Supplemental material, sj-docx-1-jbr-10.1177_07487304221082448 for Adaptation of Drosophila melanogaster to Long Photoperiods of High-Latitude Summers Is Facilitated by the ls-Timeless Allele by Peter Deppisch, Johanna M. Prutscher, Mirko Pegoraro, Eran Tauber, Christian Wegener and Charlotte Helfrich-Förster in Journal of Biological Rhythms
