# Supplementary material for: Adaptation of Drosophila melanogaster to Long Photoperiods of High-Latitude Summers Is Facilitated by the ls-Timeless Allele
Source: J Biol Rhythms. 2022 Mar 18;37(2):185–201. doi: 10.1177/07487304221082448 (PMC9008550; doi:10.1177/07487304221082448)
Supplement: sj-docx-1-jbr-10.1177_07487304221082448 – Supplemental material for Adaptation of Drosophila melanogaster to Long Photoperiods of High-Latitude Summers Is Facilitated by the ls-Timeless Allele [file sj-docx-1-jbr-10.1177_07487304221082448.docx]

**Supplemental material:**

**Table S1: Rhythmicity, circadian period and power of the different *D. melanogaster* lines in DD**

| **Line** | ***tim* polymorphism** | **n** | **% rhythmicity** | **Period (±SEM)** | **Power (±SEM)** |
| --- | --- | --- | --- | --- | --- |
| Oel 5 | *ls-tim* | 15 | 100 | 24.3 ± 0.17 | 49.1 ± 4.07 |
| Oel 6 | *s-tim* | 13 | 100 | 24.1 ± 0.11 | 28.3 ± 2.85 |
| Oel 7 | equal | 16 | 93.8 | 24.0 ± 0.11 | 28.7 ± 1.82 |
| Oel 9 | *s-tim* | 16 | 93.8 | 24.0 ± 0.13 | 35.9 ± 4.10 |
| Oel 11 | equal | 15 | 100 | 23.8 ± 0.08 | 54.4 ± 3.72 |
| Hub 5 | *s-tim* | 14 | 100 | 24.2 ± 0.07 | 49.0 ± 3.48 |
| Hub 10 | *s-tim* | 16 | 100 | 23.6 ± 0.06 | 32.6 ± 2.90 |
| Hub 33 | *ls-tim* | 31 | 100 | 23.9 ± 0.06 | 45.3 ± 3.26 |
| Hub 17 | equal | 16 | 93.3 | 24.2 ± 0.01 | 34.0 ± 2.90 |
| Hub 18 | equal | 16 | 87.5 | 24.2 ±0.10 | 46.7 ± 3.32 |
| Hub 20 | *s-tim* | 15 | 93.3 | 24.2 ± 0.11 | 45.0 ± 3.31 |
| Hub 27 | equal | 16 | 93.8 | 24.2 ± 0.15 | 41.5 ± 4.27 |
| Hub 28 | *s-tim* | 14 | 92.9 | 24.2 ± 0.09 | 42.1 ± 3.92 |
| Hub 11 | *ls-tim* | 15 | 100 | 24.1 ± 0.09 | 47.8 ± 6.06 |
| Hub 37 | equal | 14 | 100 | 24.0 ± 0.15 | 32.0 ± 2.77 |
| Hub 38 | *s-tim* | 15 | 93.3 | 24.1 ± 0.13 | 28.4 ± 2.65 |
| Lei 1 | *s-tim* prevailing | 15 | 100 | 23.9 ± 0.06 | 42.8 ± 2.68 |
| Lei 6 | *ls-tim* | 30 | 96.7 | 24.1 ± 0.06 | 41.9 ± 2.43 |
| Lei 7 | equal | 31 | 100 | 23.7 ± 0.07 | 36.7 ± 1.83 |
| Lei 8 | *ls-tim* | 32 | 90.6 | 23.6 ± 0.11 | 38.3 ± 2.69 |
| Lei 11 | equal | 30 | 100 | 24.1 ± 0.06 | 48.7 ± 2.07 |
| Lei 12 | *ls-tim* prevailing | 16 | 100 | 24.0 ± 0.06 | 38.6 ± 4.47 |
| Lei 14 | *ls-tim* | 31 | 100 | 23.6 ± 0.06 | 45.9 ± 2.12 |
| Lei 19 | equal | 16 | 100 | 23.9 ± 0.07 | 49.1 ± 4.17 |
| Lei 29 | *s-tim* | 31 | 96.8 | 24.2 ± 0.05 | 52.0 ± 2.26 |
| Lei 33 | *ls-tim* prevailing | 32 | 96.9 | 24.1 ± 0.09 | 32.3 ± 2.27 |
| Lei 38 | *s-tim* | 32 | 87.5 | 23.3 ± 0.08 | 28.5 ± 1,82 |
| Lei 39 | *s-tim* prevailing | 15 | 100 | 24.5 ± 0.09 | 38.3 ± 2.27 |
| Lei 62 | *s-tim* | 31 | 96.8 | 24.0 ± 0.07 | 59.9 ± 2.85 |
| Kop 3 | *ls-tim* | 32 | 100 | 23.4 ± 0.05 | 54.6 ± 2,46 |
| Kop 10 | *ls-tim* | 32 | 100 | 23,4 ± 0.04 | 36.4 ± 1.85 |
| Kop 12 | *ls-tim* | 31 | 96.8 | 24.3 ± 0.09 | 36.2 ± 2.69 |
| Kop 48 | *ls-tim* | 31 | 93.5 | 23.6 ± 0.06 | 26.7 ± 1.74 |
| Tan 1 | *s-tim* | 16 | 87.5 | 23.7 ± 0.20 | 25.1 ± 1.55 |
| Acc 1 | *s-tim* | 31 | 96.8 | 23.3 ± 0.09 | 39.4 ± 2.90 |
| Acc 2 | *s-tim* | 32 | 96.9 | 24.1 ± 0.08 | 42.4 ± 2.32 |
| Acc 3 | *s-tim* | 30 | 100 | 23.0 ± 0.04 | 43.7 ± 1.67 |
| Acc 4 | *s-tim* | 31 | 96.8 | 23.4 ± 0.09 | 31.5 ± 2.01 |
| Zam 27 | *s-tim* | 31 | 93.5 | 23.5 ± 0.06 | 29.2 ± 1.92 |
| Zbw 210 | *s-tim* | 20 | 95.0 | 23.6 ± 0.11 | 31.7 ± 3.17 |
| Canton-S | *ls-tim* | 14 | 100 | 24.4 ± 0.10 | 37.2 ± 2.92 |
| Lindelbach | *s-tim* | 58 | 98.3 | 23.7 ± 0.01 | 50.7 ± 0.06 |
| Cross 1 | *s-tim* | 15 | 100 | 24.2 ± 0.11 | 40.5 ± 3.32 |
| Cross 2 | *s-tim* | 15 | 100 | 24.3 ± 0.13 | 36.7 ± 2.19 |
| Cross 3 | *ls-tim* | 13 | 100 | 23.7 ± 0.13 | 40.2 ± 3.42 |
| Cross 4 | *equal mix* | 59 | 94.9 | 23.5 ± 0.07 | 32.0 ± 1.35 |
| Cross 5 | *equal mix* | 61 | 98.4 | 24.0 ± 0.06 | 36.2 ± 1.94 |
| Cross 6 | *equal mix* | 43 | 93.0 | 24.0 ± 0.06 | 28.4 ± 1.19 |
| Cross 7 | *equal mix* | 42 | 100 | 23.7 ± 0.04 | 34.1 ± 1.67 |

**Table S2: Rhythmicity, period and power of the different *D. melanogaster* lines in LL**

| **Line** | ***tim* polymorphism** | **n** | **% rhythmicity** | **Period (±SEM)** | **Power (±SEM)** |
| --- | --- | --- | --- | --- | --- |
| Oel 5 | *ls-tim* | 15 | 75 | 22.7 ± 0.43 | 27.0 ± 3.96 |
| Oel 6 | *s-tim* | 15 | 13.3 | 27.2 ± 1.16 | 19.1 ± 2.25 |
| Oel 7 | equal | 16 | 68.8 | 23.3 ± 0.59 | 27.1 ± 2.59 |
| Oel 9 | *s-tim* | 16 | 12.5 | 26.7 ± 1.55 | 19.1 ± 0.08 |
| Oel 11 | equal | 16 | 18.8 | 23.0 ± 1.18 | 23.9 ± 5.22 |
| Hub 5 | *s-tim* | 15 | 13.3 | 25.9 ± 3.25 | 18.2± 0.39 |
| Hub 10 | *s-tim* | 16 | 6.3 | 28.5 ± 0.00 | 17.5 ± 0.00 |
| Hub 11 | *ls-tim* | 32 | 43.8 | 22.4 ± 0.54 | 32.1 ± 4.89 |
| Hub 17 | equal | 16 | 6.3 | 28.1 ± 0.00 | 25.9 ± 0.00 |
| Hub 18 | equal | 16 | 18.8 | 24.1 ± 2.39 | 15.0 ± 2.39 |
| Hub 20 | *s-tim* | 15 | 0 | - | - |
| Hub 27 | equal | 16 | 56.3 | 24.6 ± 0.85 | 24.6 ± 2.57 |
| Hub 28 | *s-tim* | 16 | 12.5 | 21.7 ± 0.30 | 17.2 ± 0.44 |
| Hub 33 | *ls-tim* | 16 | 31.3 | 21.9 ± 0.53 | 17.9 ± 0.41 |
| Hub 37 | equal | 16 | 18.8 | 20.4 ± 1.02 | 13.9 ± 1.46 |
| Hub 38 | *s-tim* | 16 | 12.5 | 25.0 ± 2.19 | 19.7 ± 2.65 |
| Lei 1 | *s-tim* prevailing | 15 | 7.1 | 23.9 ± 0.06 | 42.8 ± 2.68 |
| Lei 6 | *ls-tim* | 30 | 46.7 | 24.1 ± 0.06 | 41.9 ± 2.43 |
| Lei 7 | equal | 30 | 10 | 23.7 ± 0.07 | 36.7 ± 1.83 |
| Lei 8 | *ls-tim* | 29 | 37.9 | 23.6 ± 0.11 | 38.3 ± 2.69 |
| Lei 11 | equal | 29 | 24.1 | 24.1 ± 0.06 | 48.7 ± 2.07 |
| Lei 12 | *ls-tim* prevailing | 14 | 35.7 | 24.0 ± 0.06 | 38.6 ± 4.47 |
| Lei 14 | *ls-tim* | 32 | 53.1 | 23.6 ± 0.06 | 45.9 ± 2.12 |
| Lei 19 | equal | 16 | 0 | - | - |
| Lei 29 | *s-tim* | 29 | 10.3 | 24.2 ± 0.05 | 52.0 ± 2.26 |
| Lei 33 | *ls-tim* prevailing | 32 | 9.4 | 24.1 ± 0.09 | 32.3 ± 2.27 |
| Lei 38 | *s-tim* | 30 | 3.3 | 23.3 ± 0.08 | 28.5 ± 1,82 |
| Lei 39 | *s-tim* prevailing | 15 | 20 | 24.5 ± 0.09 | 38.3 ± 2.27 |
| Lei 62 | *s-tim* | 31 | 6.5 | 24.0 ± 0.07 | 59.9 ± 2.85 |
| Kop 3 | *ls-tim* | 31 | 96.8 | 23.4 ± 0.05 | 54.6 ± 2,46 |
| Kop 10 | *ls-tim* | 32 | 12.5 | 23,4 ± 0.04 | 36.4 ± 1.85 |
| Kop 12 | *ls-tim* | 32 | 6.3 | 24.3 ± 0.09 | 36.2 ± 2.69 |
| Kop 48 | *ls-tim* | 32 | 18.8 | 23.6 ± 0.06 | 26.7 ± 1.74 |
| Tan 1 | *s-tim* | 16 | 6.3 | 22.8± 0.00 | 25.3 ± 0.00 |
| Acc 1 | *s-tim* | 32 | 3.1 | 23.3 ± 0.00 | 19.4 ± 0.00 |
| Acc 2 | *s-tim* | 32 | 3.1 | 21.9 ± 0.00 | 19.2 ± 0.00 |
| Acc 3 | *s-tim* | 31 | 0 | - | - |
| Acc 4 | *s-tim* | 31 | 0 | - | - |
| Zam 27 | *s-tim* | 31 | 0 | - | - |
| Zbw 210 | *s-tim* | 29 | 13.8 | 23.6 ± 0.11 | 18.1 ± 1.62 |
| Canton-S | *ls-tim* | 15 | 53.3 | 25.8 ± 0.79 | 19.2 ± 0.74 |
| Lindelbach | *s-tim* | 30 | 0 | - | - |
| Cross 1 | *s-tim* | 16 | 6.3 | 21.1 ± 0.00 | 19.8 ± 0.00 |
| Cross 2 | *s-tim* | 16 | 6.3 | 27.2 ± 0.00 | 20.3 ± 0.00 |
| Cross 3 | *ls-tim* | 13 | 76.9 | 23.1 ± 0.79 | 19.3 ± 1.30 |

**Table S3: Timing of evening maxima and difference between morning and evening maxima of the different *D. melanogaster* lines under long photoperiods (LD16:08, LD20:04)**

| **Line** | ***tim* poly-**  **morphism** | **n** | **E peak 16:08**  **(in h after lights-on)** | **E peak 20:04**  **(in h after lights-on)** | **Δψ_M,E_ 16:08**  **(h)** | Δψ_M,E_ **20:04 (h)** |
| --- | --- | --- | --- | --- | --- | --- |
| Oel 5 | *ls-tim* | 62 | 15.42 | 16.91 | 15.12 | 16.55 |
| Oel 6 | *s-tim* | 61 | 14.13 | 13.10 | 13.81 | 12.88 |
| Oel 7 | equal | 64 | 14.89 | 16.32 | 14.65 | 16.11 |
| Oel 9 | *s-tim* | 61 | 14.56 | 13.91 | 14.26 | 13.62 |
| Oel 11 | equal | 47 | 15.44 | 15.02 | 15.04 | 14.67 |
| Hub 5 | *s-tim* | 61 | 14.74 | 13.72 | 14.42 | 13.39 |
| Hub 10 | *s-tim* | 63 | 14.64 | 14.09 | 14.26 | 13.77 |
| Hub 11 | *ls-tim* | 46 | 15.77 | 17.37 | 15.40 | 17.07 |
| Hub 17 | equal | 61 | 13.96 | 14.33 | 13.51 | 13.17 |
| Hub 18 | equal | 63 | 15.04 | 14.84 | 14.78 | 14.61 |
| Hub 20 | *s-tim* | 62 | 15.14 | 14.28 | 14.85 | 13.99 |
| Hub 27 | equal | 62 | 14.90 | 16.53 | 14.51 | 16.19 |
| Hub 28 | *s-tim* | 47 | 15.21 | 14.57 | 14.73 | 13.94 |
| Hub 33 | *ls-tim* | 61 | 14.29 | 15.04 | 14.50 | 14.80 |
| Hub 37 | equal | 64 | 14.14 | 13.34 | 13.89 | 13.98 |
| Hub 38 | *s-tim* | 62 | 14.40 | 14.29 | 14.15 | 13.93 |
| Lei 1 | *s-tim* prevailing | 30 | 14.85 | 14.86 | 14.65 | 14.59 |
| Lei 6 | *ls-tim* | 63 | 16.22 | 18.24 | 16.10 | 17.84 |
| Lei 7 | equal | 64 | 16.18 | 14.66 | 15.93 | 14.19 |
| Lei 8 | *ls-tim* | 63 | 14.81 | 16.31 | 14.41 | 15.94 |
| Lei 11 | equal | 64 | 15.07 | 16.16 | 14.79 | 15.89 |
| Lei 12 | *ls-tim* prevailing | 64 | 14.94 | 15.81 | 14.38 | 15.45 |
| Lei 14 | *ls-tim* | 63 | 15.28 | 16.84 | 15.07 | 16.57 |
| Lei 29 | *s-tim* | 64 | 14.44 | 14.17 | 14.11 | 13.91 |
| Lei 33 | *ls-tim* prevailing | 32 | 15.08 | 15.41 | 14.59 | 15.08 |
| Lei 38 | *s-tim* | 64 | 14.70 | 14.10 | 14.29 | 13.78 |
| Lei 39 | *s-tim* prevailing | 61 | 14.65 | 15.22 | 14.34 | 14.91 |
| Lei 62 | *s-tim* | 64 | 14.44 | 14.51 | 14.09 | 14.15 |
| Kop 3 | *ls-tim* | 64 | 15.38 | 16.72 | 15.12 | 16.38 |
| Kop 10 | *ls-tim* | 62 | 14.31 | 14.90 | 14.14 | 14.79 |
| Kop 12 | *ls-tim* | 64 | 14.89 | 15.37 | 14.68 | 15.12 |
| Kop 48 | *ls-tim* | 64 | 15.17 | 15.86 | 14.88 | 15.57 |
| Tan 1 | *s-tim* | 64 | 13.96 | 12.76 | 13.69 | 12.55 |
| Acc 1 | *s-tim* | 64 | 13.26 | 13.36 | 12.83 | 13.03 |
| Acc 2 | *s-tim* | 60 | 13.71 | 14.42 | 13.34 | 13.93 |
| Acc 3 | *s-tim* | 62 | 12.71 | 13.06 | 12.40 | 12.64 |
| Acc 4 | *s-tim* | 62 | 13.32 | 14.07 | 12.92 | 13.72 |
| Zam 27 | *s-tim* | 42 | 13.73 | 13.05 | 13.69 | 12.94 |
| Zbw 210 | *s-tim* | 54 | 14.43 | 13.20 | 14.02 | 12.48 |
| Canton-S | *ls-tim* | 62 | 15.52 | 17.36 | 15.17 | 17.07 |
| Lindelbach | *s-tim* | 59 | 14.58 | 14.40 | 14.38 | 14.14 |
| Cross 1 | *s-tim* | 63 | 14.73 | 14.24 | 14.49 | 13.89 |
| Cross 2 | *s-tim* | 62 | 14.39 | 13.67 | 14.18 | 13.44 |
| Cross 3 | *ls-tim* | 62 | 14.97 | 16.52 | 14.70 | 16.24 |
| Cross 4 | *equal mix* | 30 | 14.99 | 14.89 | 14.83 | 14.68 |
| Cross 5 | *equal mix* | 30 | 14.77 | 14.70 | 14.35 | 14.58 |
| Cross 6 | *equal mix* | 30 | 15.19 | 15.97 | 15.90 | 15.67 |
| Cross 7 | *equal mix* | 30 | 15.42 | 15.90 | 16.20 | 15.71 |
